# The prognostic influence of tumour budding in Western patients with stage II colorectal cancer

**DOI:** 10.3332/ecancer.2020.1130

**Published:** 2020-10-29

**Authors:** Augusto Leite Canguçu, Ediel Valério, Roberto Bonfim Pimenta Peixoto, Tiago Cordeiro Felismino, Celso Abdon Lopes de Mello, Tatiane Neotti, Vinicius Fernando Calsavara, Mariana Petaccia de Macedo, Samuel Aguiar Júnior, Rachel Riechelmann

**Affiliations:** 1 Department of Clinical Oncology, AC Camargo Cancer Center, Rua Antonio Prudente 211, Sao Paulo, SP CEP: 01509-010, Brazil; 2 Department of Pathology, AC Camargo Cancer Center, CEP: 01509-010, Brazil; 3 Department of Epidemiology and Statistical, AC Camargo Cancer Center, CEP: 01509-010, Brazil; 4 Department of Pathology, Hospital Sírio Libânes, CEP: 01308-050, Brazil; 5 Department of Surgery Oncology, AC Camargo Cancer Center, CEP: 01509-010, Brazil

**Keywords:** tumour budding, colorectal cancer, prognostic factors, adjuvant chemotherapy

## Abstract

**Background:**

Tumour budding (TB) refers to loss of tumour cohesiveness and is defined as isolated cells or a cell cluster of up to four tumour cells at the microscopic analysis. The International Tumour Budding Consensus Conference (ITBCC) in 2016 proposed a scoring system to standardise the pathology evaluation of TB in colorectal cancer (CRC) as high (H), intermediate (I) and low (L) TB.

**Objective:**

To evaluate the recurrence-free survival (RFS) of stage II CRC patients as per the ITBCC 2016 classification and associations between TB and clinical pathological features.

**Methods:**

Cases of stage II CRC undergoing surgery with available tumour tissue underwent central pathology review for TB. Prognostic factors, retrospectively retrieved from electronic medical charts, were evaluated in univariate and multivariate Cox regression analyses for RFS (primary end point).

**Results:**

Among 137 patients included, L-TB was observed in 107 (78.1%), I-TB in 21 (15.3%) and H-TB in 9 (6.6%). In a median follow-up of 69 months, the median RFS was 134 months, with 14 patients (10.2%) presenting with tumour recurrence: 10 (9.3%) with L-TB, 2 (9.5%) with I-TB and 2 (22.2%) with H-TB. Perineural invasion was more commonly seen in the H-TB group. In multivariate analysis, TB (H and I versus L; HR = 2.6; *p* = 0.059) and not receiving adjuvant chemotherapy (HR 3.7; *p* = 0.020) were independently associated with RFS. Adjuvant chemotherapy was associated longer RFS (HR = 3.7; *p* = 0.022).

**Conclusion:**

In this series of Western patients, TB grade was associated with perineural invasion and increased risk of disease relapse.

## Introduction

The main treatment for patients with stage II colorectal cancer (CRC) is surgery with adjuvant chemotherapy, which is still debatable in this setting. The recommendation to initiate adjuvant fluoropyrimidine is based on high-risk tumour pathological features that are associated with disease recurrence [1–3]. The widely accepted high-risk characteristics used to recommend adjuvant treatment for microsatellite stable tumours are extramural vascular invasion, grade 3 or poorly differentiated histology, pathological T4 stage, perforation and/or obstructive tumours, and less than 12 lymph nodes harvested [4–6]. The American Society of Clinical Oncology guidelines published in 2019 added tumour budding (TB) as a prognostic factor for pathological stage II CRC [4], but does not formally recommend to consider it alone to administer adjuvant chemotherapy.

TB represents a cluster of tumour cells in front of the tumour specimen and is already studied in several solid tumours, demonstrating a negative prognostic impact on oesophageal [7, 8], breast [9], pancreatic [10] and lung [11] carcinomas. It is considered to be the first step in cancer metastasis, where budding cells are thought to migrate through the extracellular matrix, invading lymphovascular structures and forming metastatic tumour colonies in lymph nodes and at distant sites [12]. TB classified as either present or absent was first documented in 1989 by researchers in CRC [13]. In the early 1990s, investigators showed associations between TB and poor tumour cell differentiation, positive lymphatic invasion, greater staging and distant metastasis among Japanese patients with CRC [14–16]. Then, associations between absent [17–19] or low [20–25] (less than 10 buds) TB and respective lower or higher recurrence-free survival in stage II CRC were reported.

The various studies evaluating TB so far have used different pathological classifications. Therefore, the International Tumour Budding Consensus Conference (ITBCC) gathered in 2016 to bring together pathology to standardise the pathological classification of TB. According to this consensus, TB is defined as an isolated cancer cell or a cell cluster of up four tumour cells in the invasive front and is graded according to its number in a microscopic field. TB is stratified into peritumoural budding (PTB, tumour buds at the tumour front) and intratumoural budding (ITB, tumour buds in the tumour centre). PTB can only be assessed in endoscopic or surgical resection specimens, whereas ITB can be assessed in both cancer biopsies and resection specimens [26]. Only one study evaluated TB according to the new international classification, which was conducted in an Eastern population, showing that the number of buds is associated with recurrence-free survival (RFS) [35]. Therefore, the pursuit for additional prognostic parameters remains a hot topic in CRC-related research studies. The objective was to evaluate the prognostic impact of TB according to the new published classification, as well as to relate the budding with other clinical pathological characteristics.

## Methods

### Study design

This was a retrospective, longitudinal and single-centred study based on data extracted from patients’ electronic medical charts. The study was conducted according to the Declaration of Helsinki and was approved by the Ethics and Research Committee of the institution.

### End points

The primary end point of this study was RFS by the TB group, which was defined as the time from the date of CRC surgery until the date of recurrence or death, whichever occurred first. Recurrence was determined by tumour biopsy or radiology. Isolated elevation of carcinoembryonic antigen was not a considered recurrence. The secondary end point was the relationship between TB and other prognostic factors such as age, comorbidities, primary tumour location, microsatellite instability status and systemic treatment.

### Population and eligibility criteria

All patients with CRC who underwent resection of the primary tumour at AC Camargo Cancer Centre (ACCCC), Sao Paulo, Brazil, had their clinical data entered into a prospective database. Our study population was extracted from this database and was composed of all consecutive patients operated from 2007 to 2017. We chose this time frame because the electronic medical records were implemented in 2007; we included patients up to December 2017 to allow sufficient follow-up time to evaluate disease recurrence.

Inclusion criteria were histological diagnosis of colon, rectosigmoid or upper (intraperitoneal) adenocarcinoma of pathological stage II (AJCC v6 [27] and v7 [28]). Exclusion criteria were absence of sufficient anatomopathological material for review and absence of follow-up at the ACCCC after the surgical procedure. The following clinical pathological variables were collected: gender, date of birth, date of diagnosis, ECOG, comorbidities, medications in use, family history of neoplasia, smoking history, histological differentiation, primary tumour location, status T, number of resected lymph nodes, lymphovascular invasion, perineural invasion, preoperative carcinoembryonic antigen human (CEA) level, postoperative CEA level, RAS status, presence of microsatellite instability (MSI), systemic therapy used, number of buds evaluated in the surgical piece, poorly different clusters, body mass index, date of last follow-up, date and site of recurrence and date of death, if occurred.

### TB evaluation

Paraffin-embedded tumour tissues were retrieved and revised by two independent pathologists who evaluated TB that was considered as single tumour cells or clusters of up to four cells in the advancing tumour front as per the ITBCC 2016 [26]. TB was assessed in one hotspot at the invasive tumour front in an area measuring 0.785 mm^2^ which corresponds to the 20x field in the microscope used in this study with an eyepiece field number of 20 mm and a normalisation factor of 1.0.

**Figure 1.**
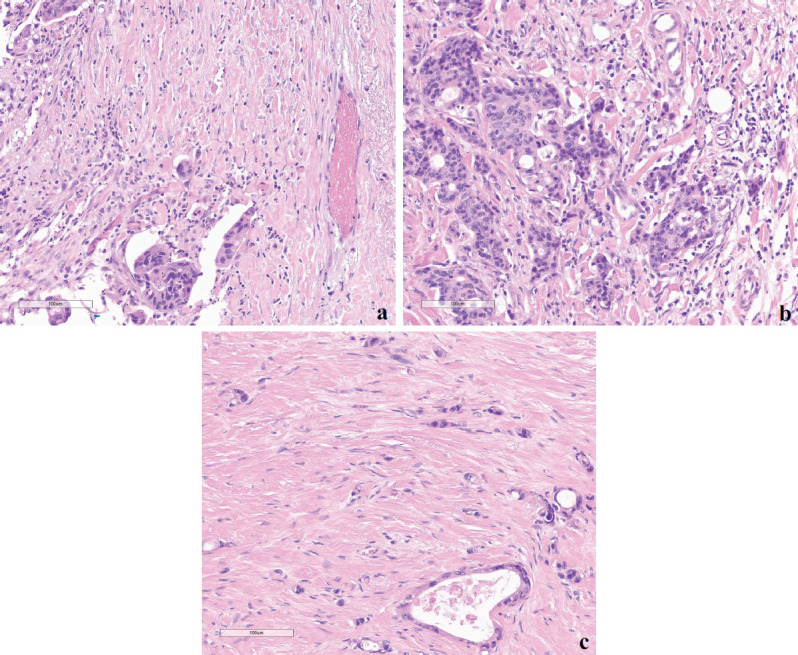
Examples of different TB grades (hotspot = 0.785 mm^2^) at the invasive front of colorectal cancer based on ITBCC 2016. (a): Low-TB; (b): Intermediate-TB and (c): High-TB.

TB was classified as less than five, five to nine, and ten or more budding foci with low (L-TB), intermediate (I-TB) and high (H-TB) grades, respectively (Figure 1). No cytokeratin stains were used to determine the hotspots or to count the number of buds. The pathologists also revised other pathological features such as histological grade, perineural and vascular/lymphatic invasions and number of lymph nodes in the specimen. Disagreements were managed by discussion and a consensus was achieved in all cases.

### Statistical analysis

The baseline patients’ characteristics were reported as absolute and relative frequencies for qualitative variables and as median, minimum and maximum for quantitative variables. The association between qualitative variables was evaluated by chi-square (χ^2^) test or Fisher’s exact test, as appropriate. The non-parametric Kruskal–Wallis test was applied to compare the distribution of quantitative variables across TB groups (low, intermediate and high). RFS was estimated using the Kaplan–Meier estimator and the log-rank test was used to compare the RFS among groups. The Cox semiparametric proportional hazards model was fitted to describe the relationship between RFS and the covariates. The assumption of proportional hazards was assessed based on the so-called Schoenfeld residuals [29, 30]. There was evidence that covariates had a constant effect over time in all cases.

To investigate the independent influence of TB and other prognostic variables on RFS, a simple Cox model was used. Ten pre-specified prognostic variables were tested: number of lymph nodes examined, tumour differentiation (poor versus well/moderate), T stage (pT4 versus pT3), vascular/lymphatic invasion, perineural invasion, immunohistochemistry expression of mismatch repair proteins (deficient versus proficient), Charlson comorbidity index, age (continuous), receipt of adjuvant treatment and TB classification. Because of the small number of events, we grouped I-TB and H-TB and compared the prognosis of this group with L-TB. Pathological T4 was forced into the multivariable model independently of its result in the univariate analysis because of its known worse prognosis.

Statistical analyses were carried out using R software version 3.5 (R Foundation for Statistical Computing, Vienna, Austria). The significance level was two-tailed and fixed at 5% for all tests.

## Results

### Clinical pathological features

During the study period (January 2007 to December 2017), 189 patients were screened and 137 were eligible for the study. The exclusion reasons were 38 patients lacked sufficient pathological material for review and 14 were not followed-up at our institution after CRC surgery.

In summary, the male gender was more prevalent with 77 cases (56.2%). The median age at diagnosis was 61 (25–93) years. Seventy-one (51.8%) had left-sided tumours. One hundred and thirteen cases (82.5%) had pT3 CRC. The median of resected lymph nodes was 21; 13 (9.5%) patients had positive lymphatic/vascular and 15 (10.9%) had perineural invasions in their tumours. Fifty patients (36.5%) received adjuvant systemic treatment. One hundred and seven patients had TB (78.1%) classified as L-TB, 21 (15.3%) as I-TB and 9 (6.6%) as H-TB.

In all three groups, there was a prevalence of left-side primary tumours. The proportion of T4 was similar across groups, although being numerically higher in H-TB. Perineural invasion was significantly associated with H-TB, with six positive cases (5.6%) in the L-TB group, four (19.0%) in the I-TB group and five (55.6%) in the H-TB group (*p* < 0.001).

Microsatellite instability was evaluated in 82 patients (59.8% of the sample) and 12 patients presented a deficient profile. Adjuvant systemic therapy was administered to 50 (36.5%) patients and it was more commonly administered to patients whose tumours had H-TB (*p* = 0.042) (Table 1).

In the median follow-up of 69 months (95% Confidence Interval (CI): 64–73), 14 patients (10.2%) experienced disease recurrence. The rate of recurrence was 9.3%, 9.5% and 22.2% in the L-TB, I-TB and H-TB groups, respectively. The organs mostly involved by metastasis were liver, lung and lymph nodes in the L-TB; lung in the I-TB; and locoregional and lymph node in the H-TB. (Table 2).

### Prognostic factors for RFS

**Figure 2.**
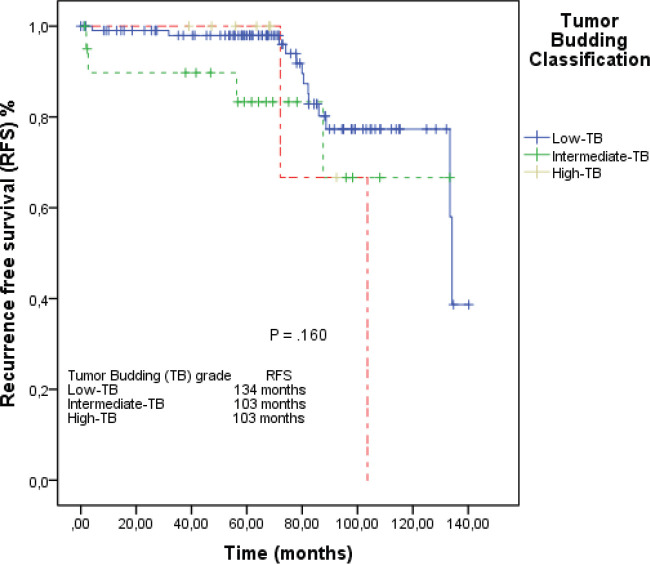
RFS by the Kaplan–Meier estimator: low-TB versus intermediate-TB versus high-TB.

In the median follow-up time of 69 months, the median of RFS in the overall population was 134 months (95% CI: 103–164). In the L-TB group, median RFS was 134 months (95% CI: 132–135). In the I-TB and H-TB groups, the median RFS for both was 103 months (95% CI: 85–129; 95% CI: 69–116, respectively; Figure 2).

**Figure 3.**
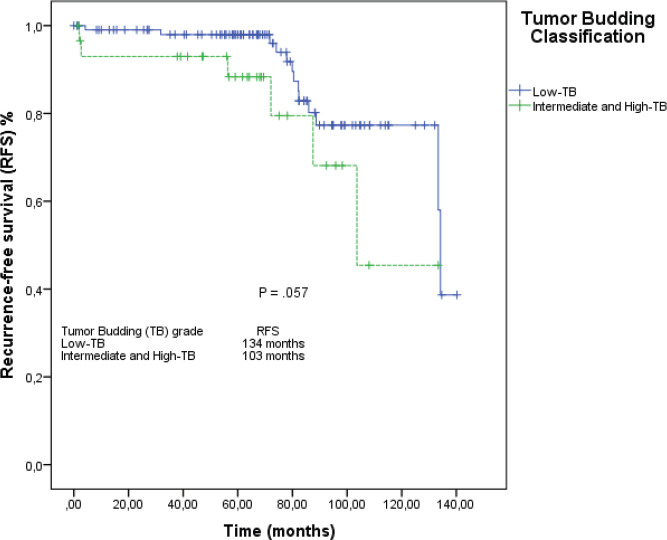
RFS by the Kaplan–Meier estimator: low-TB versus intermediate-TB and high-TB.

When we grouped I-TB and H-TB, the median RFS was 103 months (95% CI: 84–122), versus 134 months in the L-TB group (95% CI: 132–135). The RFS rate at 5 years for L-TB, I-TB and H-TB groups were, respectively, 98%, 88.3% and 66.7% (*p* = 0.160). When we grouped I-TB and H-TB versus L-TB, the RFS rates at 5 years were 88.3% versus 98%, respectively (*p* = 0.057; Figure 3).

The multivariable Cox model revealed that the I-TB and H-TB versus L-TB (HR = 2.6; 95% CI: 0.9–7.1; *p* = 0.05) and the lack of adjuvant chemotherapy receipt (HR = 3.7; 95% CI: 1.2 to 11.3; *p* = 0.02) were independently associated with RFS (Table 3).

## Discussion

In this retrospective series of consecutive patients with stage II CRC using the new pathological ITBCC, upper TB grades had a 2.6 higher odds of experiencing disease recurrence compared to the L-TB. While patients whose CRC presented H-TB also had more often tumours with perineural invasion, H-TB was not associated with other prognostic features, such as pT4 tumours.

Our data are consistent with data from previous publications that identified TB as a prognostic factor for RFS in stage II CRC [15, 18, 23, 26, 31]. However, to the best of our knowledge, this study represents the largest TB series in Western patients with stage II CRC according to the new international TB classification. The SACURA trial [35], published in 2018, also evaluated TB as per the ITBCC 2016 classification. They examined the influence of TB on tumour-related outcomes among 991 Japanese stage II CRC patients. Differently from our findings, the SACURA study observed that T4 disease and positive vascular/lymphatic invasion were associated with H-TB. Perineural invasion evaluation was not carried out in the SACURA study. In our study, positive perineural invasion was associated with TB degree in patients with H-TB. In both SACURA and our study, there was no association between sidedness and TB. Likewise tumour recurrence occurred in nearly 25% and 22% of patients with H-TB in SACURA and our study, respectively, in a similar follow-up period (60 and 69 months, respectively). Regarding survival, SACURA patients in the I-TB and H-TB groups achieved an RFS at 5 years of 85.1% and 74.4%, respectively. In our population, the grouping of I-TB and H-TB patients achieved a 5-year RFS rate of 66.7%. Such differences in results between our sample of Western patients and the Japanese study may reflect unknown differences in prognosis between populations or because of our smaller sample.

The evaluation of TB in 979 patients from the QUASAR study, which investigated the role of adjuvant 5-FU in stage II CRC, was carried out [36]. Although not using the new ITBCC 2016 classification, in a 120 months follow-up, there was a 35% of disease recurrence in patients with a high degree of budding (high degree defined as above 10 buds per 1.23/mm³ field) and according to the study, there was a non-significant trend towards increasing chemotherapy efficacy with increasing budding counts (*p* = 0.12). Given the small number of events of recurrence in our study, we could not examine whether adjuvant chemotherapy decreased recurrence in patients with I-TB and H-TB.

TB is a well-documented histological phenomenon in CRC and is considered to be the first step in cancer metastasis [22, 25, 30]. Studies comparing the molecular profile of budding cells with tumour cells showed differences in relation to the expression of cell adhesion proteins [32, 33]. A study using RNA sequencing showed the exchange of the epithelial molecular type for mesenchymal in budding regions when compared to other areas of the tumour, indicating that the molecular background is not constant throughout the tumour [34].

Besides TB, several strategies are being tested as possible prognostic and predictive factors of recurrence in stage II CRC, precisely to help the decision of the adjuvant chemotherapy in this setting [37, 38], such as gene platforms, molecular evaluations of circulating tumour DNA, immunohistochemistry expression of CDX2 and immunoscore, among others.

A study evaluating the clinical applicability of ColoPrint, a gene platform that assesses the frequency of oncogenes and tumour suppressor genes, in patients with stage II CRC is ongoing (NCT00903565). Others observational studies in progress assess the use of microRNA by RT-PCR after curative surgery [39, 40] and the expression of caudal-type homeobox transcription factor (CDX-2) in colon epithelial tissues. CDX2-negative tumours are often associated with several adverse prognostic variables (e.g., advanced stage, poor differentiation, vascular invasion, *BRAF* mutation and CIMP-positive status) and lower DFS [41–43]. The immunoscore is derived from a measure of CD3-positive and CD8-positive cell densities in the tumour centre and invasive margin, and is considered a prognostic biomarker in colon cancer. In an immunoscore study, the prognostication was shown in training and validation sets and in analyses of time to recurrence (the primary end point), disease-free survival and overall survival [44].

Our study is limited by its retrospective design, although selection bias may have been minimal because we enrolled all consecutive patients from our colorectal surgical database. During the study period, staging and treatment were adjusted due to international consensus. The patients were staged based on different versions of the AJCC [24, 25], but we believe that this did not impact the analysis of the study as a whole since they did not have major changes between the sixth and seventh editions. The microsatellite instability profile was carried out in only 40.2% of the patients because its assessment became standard in recent years and unfortunately we could not evaluate it during our pathological review due to budget restrictions. Positive aspects of our study include a consecutive sample of patients, a pathological review of all cases and it brings important data on TB in Western CRC patients.

## Conclusion

In conclusion, I-TB and H-TB were associated with worse RFS compared to L-TB in patients with stage II CRC. H-TB was also associated with a higher rate of perineural invasion. However, the recommendation of adjuvant chemotherapy based on TB should be evaluated in larger studies.

## Conflict of interest

The authors declare that they have no financial competing interests.

## Funding statement

The authors declare that this research did not receive any funding or grants.

## Authors’ contributions

RR planned the review. ALC, RR, TCF and CALM did the initial literature research. SAJ did additional searches. EV, RBPP, TN and MPM performed the anatomopathological review. VFC contributed to statistical analyses. ALC contributed to data extraction and drafted the initial manuscript. All authors contributed to several rounds of revisions. All authors read and approved the final version of the manuscript.

## Figures and Tables

**Table 1.** TB and pathological clinical features.

	Grade of TB			
**Parameters**	**Low-TB** ***n* = 107 (78.1%)**	**Intermediate-TB ** ***n* = 21 (15.3%)**	**High-TB ** ***n* = 9 (6.6%)**	***p***
**Sex**				
Male	60 (56.1)	11 (52.4)	6 (66.7)	0.769
Female	47 (43.9)	10 (47.6)	3 (33.3)	
**Age (min–max)**	61 (25–91)	61 (39–93)	57 (37–72)	0.192
**Charlson comorbidity index**				
≥ 6.0	21 (19.6)	6 (29.6)	0 (0.0)	0.227
< 6.0	86 (80.4)	15 (71.4)	9 (100.0)	
**Tumour location**				
Right-sided colon	31 (29.0)	5 (23.8)	2 (22.2)	0.924
Left-sided colon	54 (50.5)	11 (52.4)	6 (66.7)	
Rectum	22 (20.6)	5 (23.8)	1 (11.1)	
**T stage**				
T3	89 (83.2)	17 (81.0)	7 (77.8)	0.768
T4	18 (16.8)	4 (19.0)	2 (22.2)	
**Extension of LN dissection (min–max)**	23 (8–98)	22 (14–78)	20 (7–49)	0.976
**Lymphatic vascular invasion**				
Negative	98 (91.6)	17 (81.0)	9 (100.0)	0.237
Positive	9 (8.4)	4 (19.0)	0 (0.0)	
**Perineural invasion**				
Negative	101 (94.4)	17 (81.0)	4 (44.4)	< 0.001
Positive	6 (5.6)	4 (19.0)	5 (55.6)	
**Tumour cells differentiation**				
G1	5 (4.7)	0 (0.0)	0 (0.0)	0.710
G2 and G3	102 (95.3)	21 (100.0)	9 (100.0)	
**Preoperative CEA, ng/m**				
≤4.0	54 (56.8)	6 (40.0)	3 (33.3)	0.252
>4.0	41 (43.2)	9 (60.0)	6 (66.7)	
**MSI status**				
Proficient	54 (83.1)	10 (90.9)	6 (100.0)	0.730
Deficient	11 (16.9)	1 (9.1)	0 (0.0)	
**Adjuvant treatment**				
Performed	40 (37.4)	4 (19.0)	6 (66.7)	0.042
Not Performed	67 (62.6)	17 (81.0)	3 (33.3)	

Note 1: Data are No. (%) unless otherwise indicatedAbbreviations: CEA, carcinoembryonic antigen; LN, lymph node; MSI, microsatellite instability

**Table 2.** Sites of disease recurrence by TB group.

Sites of recurrence	Low-TB n = 107 (78.1%)	Intermediate-TB n = 21 (15.3%)	High-TB n = 9 (6.6%)
Locoregional	0 (0%)	0 (0%)	1 (11.1%)
Lymph nodes	2 (1.9%)	0 (0%)	1 (11.1%)
Liver	6 (5.6%)	0 (0%)	0 (0%)
Lung	2 (1.9%)	2 (9.5%)	0 (0%)
Recurrence (Total)	10 (9.3%)	2 (9.5%)	2 (22.2%)

Note 1: Data are No. (%) unless otherwise indicatedAbbreviations: TB: tumour budding

**Table 3.** Univariable and multivariable Cox regression model for RFS.

		Univariable		Multivariable	
**Parameters**	**No.**	**HR (95% CI)**	***p***	**HR (95% CI)**	***p***
**Tumour Budding**					
Low-TB	107	1		1	
I + H-TB	30	2.54 (0.93–6.90)	0.067	2.61 (0.96–7.11)	0.059
**Charlson comorbidity index**					
≤ 6.0	110	1		1	
> 6.0	27	3.48 (1.20–10.0)	0.021	2.47 (0.81–7.46)	0.109
**Tumour location**					
Right-sided colon	38	1			
Left-sided colon	71	0.98 (0.34–2.82)	0.978		
Rectum	28	0.34 (0.03–3.05)	0.338		
**Age**	137	1.03 (0.99-1.08)	0.091	1.01 (0.96–1.06)	0.695
**T stage**					
T3	113	1		1	
T4	24	0.84 (0.24–2.93)	0.787	1.56 (0.35–6.93)	0.553
**Lymphatic vascular invasion**					
Negative	124	1			
Positive	13	0.38 (0.05–2.93)	0.357		
**Perineural invasion**					
Negative	122	1			
Positive	15	1.14 (0.32–4.01)	0.832		
**Tumour cells differentiation**					
G2 and G3	132	1			
G1	5	0.78 (0.09–6.46)	0.821		
**MSI status**					
Proficient	70	1			
Deficient	12	0.58 (0.07–4.57)	0.613		
**Adjuvant treatment**					
Performed	87	1		1	
Not Performed	50	0.27 (0.09–0.82)	0.022	3.73 (1.22–11.3)	0.020

Note 1: Data are No. (%) unless otherwise indicatedAbbreviations: HR, hazard ratio; CI, confidence interval; LN, lymph node; MSI, microsatellite instability; TB, tumour budding * Only 82 patients with MSI values were analysed
